# A Novel Tongue-Based Tumor With an RREB1-MRTFB Fusion: Variant Rhabdomyosarcoma or Aggressive Variant of Ectomesenchymal Chondromyxoid Tumor

**DOI:** 10.7759/cureus.33187

**Published:** 2022-12-31

**Authors:** Nuttavut Sumransub, Emil Racila, Paari Murugan, Conner O'Brien, Shelly Marette, Amy Lassig, Keith M Skubitz

**Affiliations:** 1 Medicine, University of Minnesota, Minneapolis, USA; 2 Laboratory Medicine and Pathology, University of Minnesota, Minneapolis, USA; 3 Laboratory Medicine and Pathology, University of Minnesota, MInneapolis, USA; 4 Oncology, Invitae, San Francisco, USA; 5 Radiology, University of Minnesota, Minneapolis, USA; 6 Otolaryngology, University of Minnesota, Minneapolis, USA

**Keywords:** genetics, translocation, ectomesenchymal chondromyxoid tumor, rreb1-mrtfb, gene fusion, sarcoma, rhabdomyosarcoma

## Abstract

The presence of a *FOXO1 *fusion in a tumor is one of the most important prognostic factors in rhabdomyosarcoma. Most histologically defined alveolar rhabdomyosarcomas bear a *FOXO1* fusion. We discuss a case that was initially thought to be a rhabdomyosarcoma but was later discovered to have an *RREB1-MRTFB* fusion. This fusion has never been reported in rhabdomyosarcoma but typically characterizes ectomesenchymal chondromyxoid tumor (ECT), a neoplasm with typically rather benign behavior. In this article, the authors discussed whether this patient’s aggressive presentation represents a variation of ECT or an unusual case of rhabdomyosarcoma.

## Introduction

Rhabdomyosarcoma is the most common soft tissue sarcoma in children and represents about half of pediatric soft tissue sarcomas; however, it is rare in adults [1,2]. Since its original description, which was based on morphologic criteria, it was grouped into four subtypes including embryonal, alveolar, botryoid, and pleomorphic. These subtypes were generally associated with different clinical biologies [2]. Subsequently, immunomarkers indicating muscle differentiation led to modifications in diagnosis and are commonly used in diagnosis. As molecular genetics has evolved, the most recent WHO classification includes four subtypes of rhabdomyosarcoma: embryonal, alveolar, spindle cell/sclerosing, and pleomorphic [3]. Embryonal rhabdomyosarcoma frequently occurs in the head and neck, genitourinary tract, and bile duct regions, while alveolar rhabdomyosarcoma is more commonly seen in older children and adolescents and typically occurs in the extremities [4]. Pleomorphic rhabdomyosarcoma typically occurs in adults, though it can be seen in children with an underlying genetic predisposition; these tumors typically contain complex genomic changes and are viewed by some as similar to undifferentiated pleomorphic sarcomas [5]. It is important to note that a number of tumors can undergo rhabdomyoblastic differentiation but are not true rhabdomyosarcoma [4]. Treatment with surgery, radiation, and chemotherapy is commonly used to treat rhabdomyosarcoma [1].

One of the most important discriminators in rhabdomyosarcoma is whether the tumor is a *PAX-FOXO1 *fusion-positive or fusion-negative rhabdomyosarcoma [6]. Most alveolar rhabdomyosarcoma defined histologically bear a *FOXO1 *fusion. Spindle cell/sclerosing rhabdomyosarcoma histology is generally associated with an *MYOD1 *mutation, and a *VGLL2-NCOA2* fusion can be seen in a congenital subtype [7,8]. In contrast, embryonal rhabdomyosarcoma has a more varied genetic landscape commonly associated with complex chromosomal changes. Most of the *FOXO1 *fusion-positive tumors include either a *PAX3-FOXO1* or a *PAX7-FOXO1* fusion, and the presence of a *FOXO1* fusion is an important marker of tumor biology. Fusion-positive alveolar rhabdomyosarcoma is generally worse than the so-called fusion-negative alveolar rhabdomyosarcoma or embryonal rhabdomyosarcoma, although fusion-negative alveolar rhabdomyosarcoma is commonly approached in a way similar to embryonal rhabdomyosarcoma [9,10].

In this article, we discuss a case initially which was classified as rhabdomyosarcoma, mostly due to its apparent myogenic differentiation, but the subsequent mutational analysis found an *RREB1-MRTFB* fusion. This fusion has never been reported in rhabdomyosarcoma but typically characterizes ectomesenchymal chondromyxoid tumor (ECT), a recently described mesenchymal tumor with a typically benign clinical course [11]. Whether this patient’s aggressive presentation represents a variation of ECT or an unusual case of rhabdomyosarcoma is discussed.

## Case presentation

**Figure 1:**
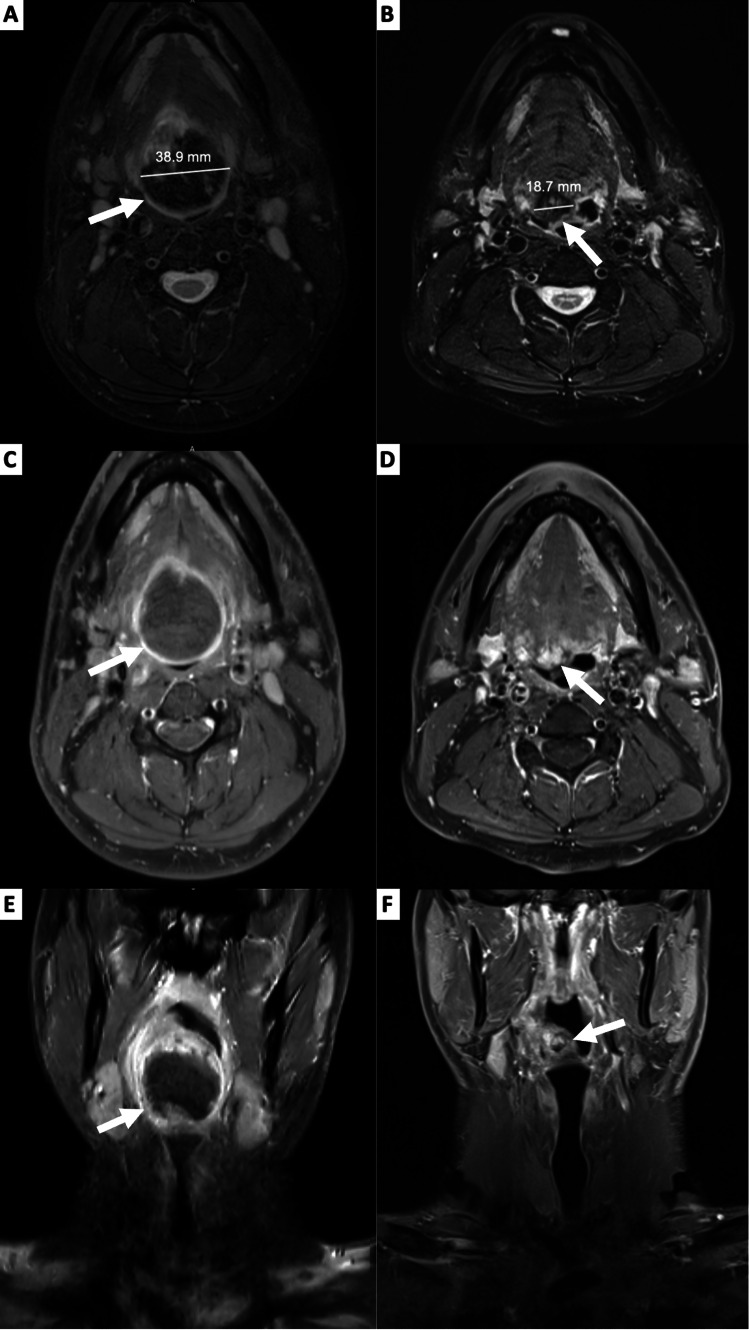
MRI images before and after chemotherapy Panels A, C, and E: The left column shows MRI images before chemotherapy. Panels B, D, and F: The right column shows MRI images after chemotherapy. (A and B) Axial T2 fat saturation images, (C and D) axial T1 fat saturation post-gadolinium contrast images, and (E and F) coronal T1 fat saturation post-gadolinium contrast images. The pretreatment images show a heterogenous mass located in the right paramedian base of the tongue with thickened peripheral enhancement (~3.9 cm x 4.4 cm). The postchemotherapy images show a smaller mass with heterogenous internal enhancement (~1.8 cm x 1.8 cm).

A 26-year-old man with no significant past history presented with hemoptysis one day prior to admission. He had a one-week history of increasing sore throat and voice change and had been spitting up blood throughout the day of admission. His family history was unremarkable. He had no history of smoking, alcohol use, or recreational drug use. On examination, he had signs of hypovolemic shock with hypotension (BP 60/26), altered mental status, elevated lactate (5.8 mmol/L), and acute renal failure with a creatinine of 1.32 mg/dL. He was treated with two liters of IV fluid, two units of packed red cells, and 1 gram of tranexamic acid IV in the emergency department. Tranexamic acid inhibits the conversion of plasminogen to plasmin, thereby exerting an anti-fibrinolytic effect. Clinically, he stopped spitting up blood after stabilization. After hemodynamic stabilization, he underwent a direct laryngoscopic examination; this revealed a smooth, soft, reddish mass on the right side of the oropharyngeal tongue. No active bleeding was seen at the time of the laryngoscopy. A pre-chemotherapy CT scan of the neck demonstrated a heterogeneously enhancing mass measuring 4.9 cm x 3.9 cm x 5.1 cm at the base of the tongue and lingual tonsils extending from the tip of the soft palate down to the epiglottic vallecula (Figure 1).

**Figure 2:**
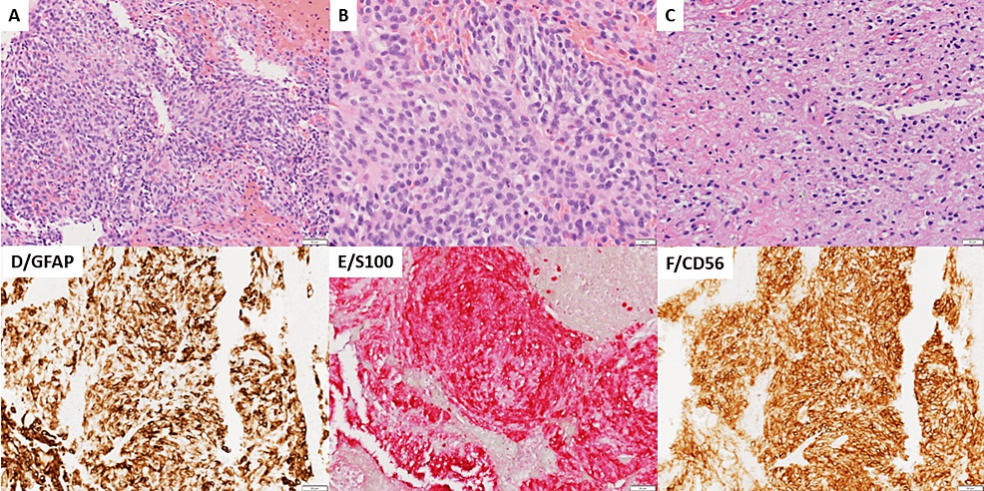
Ectomesenchymal chondromyxoid tumor histology before and after therapy ECT is a cellular neoplasm with rather bland cytologic and nuclear features. At most mild atypia may be observed in a subgroup of cells. The cells are round, ovoid, or spindle-shaped and form poorly defined fascicles with vague interlacing architecture. Mitoses are rare and, if present, not atypical. There is no tumor necrosis before chemotherapy. Tumor histology biopsy specimen images before therapy are shown in Panels A (200X) and B (400X). After therapy, treatment response is associated with cellular shrinking and necrosis (Panel C, 400X). Chondromyxoid stroma, if present, is patchy and not always well represented. The blood extravasation seen in this case is likely procedural and not a native feature of the neoplasm. Immunohistochemistry testing demonstrated the expression of GFAP (Panel D), S100 (Panel E), and CD56 (Panel F) in most neoplastic cells. ECT: Ectomesenchymal chondromyxoid tumor; GFAP: Glial fibrillary acidic protein.

There was compression of the oropharynx and displacement of the epiglottis. Biopsy was consistent with rhabdomyosarcoma with positive immunohistochemical stains for myogenin, MyoD1, S-100, synaptophysin, CD56, vimentin, actin, desmin, and glial fibrillary acidic protein (GFAP), as shown in Figure 2.

Immunohistochemical staining for Ki-67, a cellular proliferation marker, was moderately increased at 10%. Positron emission tomography-computed tomography (PET-CT) revealed an exophytic, hypermetabolic (SUV_max_: 7.3) tumor arising from the tongue base, filling in the vallecula and abutting the suprahyoid epiglottis with no distant metastasis. There was intense fludeoxyglucose (FDG) uptake in symmetric nasopharyngeal adenoidal thickening and slightly enlarged FDG-avid bilateral level 2 lymph nodes.

The patient received chemotherapy with cyclophosphamide, doxorubicin, and vincristine (CAV) alternating with ifosfamide and etoposide (IMV) for five cycles. Restaging MRI showed a residual, heterogeneous mass of approximately 2 cm x 1.3 cm in size, which was centered in the tongue base with no lymph node or distant metastasis (Figure 1). He then underwent a robotic-assisted tongue base resection. Pathologic examination of the resection specimen was consistent with residual embryonal rhabdomyosarcoma, with treatment effect, 60% tumor necrosis, and negative margins (Figure 2). No *FOXO1 *fusion was detected by fluorescence in situ hybridization (FISH) assay in the resection specimen.

The patient received postoperative proton radiation therapy (3600 cGy in 20 fractions) and two cycles of CAV/IMV. Follow-up MRI showed no evidence of disease with the most recent follow-up at two years after completion of the treatment. The tissue was later sent for next-generation sequencing (NGS) using the Sema4 platform (Sema4, Connecticut, USA), which confirmed no *FOXO1* fusion. The study detected no microsatellite instability, low tumor mutational burden (0.06 Mut/MB), and an *RREB1-MRTFB* fusion.

## Discussion

We describe the case of a patient with a very aggressive clinical course that was felt to be a *FOXO1*-negative rhabdomyosarcoma and was treated for that condition. The patient presented with acute onset of voice changes and massive hemoptysis leading to hypovolemic shock. Later, the genetic analysis demonstrated an *RREB1-MRTFB* fusion. The majority of alveolar rhabdomyosarcoma cases are associated with a characteristic chromosomal translocation involving a fusion between *PAX3 *or *PAX7 *with *FOXO1*, which is associated with more aggressive behavior and poorer prognosis [12]. Embryonal rhabdomyosarcoma with no *PAX-FOXO1* fusion is viewed as a distinct biological subtype. The tumor biology and outcome are similar between fusion-negative alveolar rhabdomyosarcoma and embryonal rhabdomyosarcoma; however, they are significantly different from fusion-positive alveolar rhabdomyosarcoma [10]. A comprehensive genetic analysis in 147 rhabdomyosarcoma performed in one study also identified recurrent genetic alteration in multiple genes, such as *CTNNB1*, *FBXW7*, and *BCOR*, as well as gain-of-function mutation of genes in the RAS pathway, including *RAS*, *PIK3CA*, and *FGFR4 *[6]. No mutations were present in any of these genes in our case.

The predicted fusion protein found in this neoplasm contains the N-terminal zinc finger domain of *RREB1* with the C-terminal transcriptional activation domain and leucine zipper-like domain from *MRTFB *[13]. The *RREB1-MRTFB* fusion has never been reported in rhabdomyosarcoma but has been identified in 90% of ECT, a mesenchymal tumor with generally benign behavior which is found almost exclusively on the anterior dorsal tongue and stains positively for smooth muscle actin, desmin, and myogenin [11]. ECT was originally described as a benign intraoral tumor typically presenting as a small painless mass on the anterior dorsal surface of the tongue; however, the course can vary with various durations of symptoms reported from weeks to up to 10 years [14,15]. Siegfried described an oropharyngeal tumor bearing the *RREB1-MRTFB* fusion with no tongue involvement in a 53-year-old man [13], and subsequent reports have described cases involving other sites outside the tongue including the mandible [16]. A recent report described two mediastinal mesenchymal tumors in adult women that also bore this fusion [17]. A recent abstract reported a case of ECT that appeared to show progression to a poorly differentiated sarcoma, suggesting transformation to a more aggressive malignancy [18]. In this case, the post-treatment specimen had a low mitotic rate, but 60% of the tumor showed necrosis with treatment effect. It is possible that a higher “mitotic index” component of the tumor was killed, and a less mitotically active component remained in the resection specimen. Such a phenomenon would be compatible with the development of a higher-grade malignancy in a previously lower-grade tumor. The biopsy specimen was of necessity a small sample, thereby limiting definitive conclusions on pre- and post-treatment mitotic rates.

Given the tumor location in our case and the clinical presentation, it is possible that this patient’s tumor may be a biologic variant of ECT with more aggressive biology than classic ECT, based on its apparent rate of growth. Alternatively, this case may represent an unusual variant of rhabdomyosarcoma.

## Conclusions

This is the first report on *RREB1-MRTFB* fusion in rhabdomyosarcoma. The location of the tumor and presentation could represent an atypical rhabdomyosarcoma or a biologic variant of a different mesenchymal tumor, ECT. This case highlights the importance of NGS in fusion-negative rhabdomyosarcoma, especially in the head and neck. We suggest that this genetic fusion should be investigated in rhabdomyosarcoma with no *PAX-FOXO1* fusion especially if they are located in the oral cavity and tongue area to aid in the diagnosis and better clarify the biology of this disease.
